# Foraminifera as indicators of species invasion: *Ammonia confertitesta* in Long Island Sound

**DOI:** 10.1126/sciadv.adv9447

**Published:** 2025-09-03

**Authors:** Eleanor J. Goetz, Pincelli M. Hull, Johan Varekamp, Ellen Thomas

**Affiliations:** ^1^ Department of Earth and Planetary Sciences, Yale University, 210 Whitney Ave., New Haven, CT 06511, USA.; ^2^ Yale Peabody Museum, 170 Whitney Ave., New Haven, CT 06511, USA.; ^3^ Department of Earth and Environmental Sciences, Wesleyan University, 265 Church Street, Middletown, CT 06459, USA.

## Abstract

Resolving timing of the invasion of nonindigenous species is difficult in estuarine settings, due to their pervasive history of anthropogenic disturbance. Many non-native marine taxa are not documented until after they have become invasive, leaving questions about invasion timing (first introduction and lag period), geographic origin, vectors and pathways, and cause(s) of success. Foraminifera, unicellular, calcareous-shelled eukaryotes, offer a unique way of analyzing past ecosystem structure because their fossilized shells provide a window into the past, and small size and abundance enable us to document distribution over time in core samples. We use records of Foraminifera to document the timing and history of establishment of an invasive species (*Ammonia confertitesta*) in Long Island Sound, east of New York City (USA). *A. confertitesta* was rare from the mid-19th century but did not proliferate until the mid-1970s. We hypothesize that increasing propagule pressure from the rapid increase in global ship traffic and ballast water was the main factor for its success.

## Teaser

Exceptional fossil record of foraminifera in Long Island Sound reveals ballast water as the primary culprit of species invasion.

## INTRODUCTION

Estuarine habitats, including wetland systems (e.g., salt marshes), provide critical ecosystem services such as breeding and nursery habitats for diverse marine biota (*1*), filtering of pollutant-contaminated freshwater runoff, and buffering of coastlines from erosion and flooding (*2*). Estuarine ecosystems are highly susceptible to anthropogenic disturbances through climate change, urbanization, and industrialization, including pollution, habitat destruction, direct exploitation, and the introduction of invasive species (*3*, *4*). Estuaries are especially vulnerable to the introduction of non-native species because they are at the nexus of coastal urbanization, trade, and ship transport (*4*, *5*). Introduced non-native species can become invasive, which can lead to cascading ecosystem degradation, biodiversity loss, and loss of ecosystem services (*4*). The relative importance of invasive species in coastal and marine ecosystem degradation may be highly underestimated due to uncertainty surrounding the timing of introduction and invasion (*6*).

In this study, we examine the non-native foraminifer *Ammonia confertitesta* in Long Island Sound (LIS) to understand the timing and mode of its introduction and the reason(s) for its successful establishment and proliferation (*7*, *8*). LIS is the second largest estuary in the eastern United States (*9*) and has a long, complex history of anthropogenic change. It is called “The Urban Sea” because of its coastal urbanization, especially in New York City at its western end. There is a strong gradient of declining water quality, declining salinity, and increasing summer deoxygenation from the east, open to the ocean and flushed by strong tidal currents through Block Island Sound, to the west, where there is a narrow opening to the Atlantic Ocean through the East River (*10*, *11*). LIS experienced severe environmental and ecological degradation since the 17th century arrival of Europeans (*12*).

Non-native aquatic species are primarily spread by release of ballast waters (*8*, *13*, *14*) and the attachment of marine organisms to ships’ hulls [i.e., biofouling; (*15*)]. Not all introduced species persist, and even fewer become invasive [i.e., so abundant as to disrupt ecosystem functioning; (*16*, *17*)]. Whether introduced species survive, and how quickly they become established and spread aggressively [e.g., (*18*)] depends, in part, on the frequency and number of individuals introduced [i.e., propagule pressure; (*18*, *19*)]. Globally, the number of aquatic invasive species greatly increased in the mid-20th to late 20th century as shipping switched to container and bulk transports and the amount of global travel and trade increased (*20*, *21*). To be successful invaders, non-native species generally must have some evolutionary and/or ecological advantage over their native counterparts, such as a lack of natural predators, short generation times with many offspring, different or broader food preferences, and an ability to adapt quickly to their non-native environment (*22*–*24*). Ecosystems may become more susceptible to invasions when they are destabilized through changed ecological, biological, chemical, or physical states (*8*).

In LIS, factors linked to the spread and impact of invasive species (i.e., connectivity, propagule pressure, and environmental change) have a long history. Marked anthropogenic environmental change may have started with the beaver fur trade primarily from the early 17th century to mid-18th century, which altered the New England landscape along with increasing freshwater and nutrient delivery to the sound (*25*, *26*). By the second half of the 19th century (*27*), rapid population growth and industrialization in New York City led to widespread pollution of LIS. Industrial pollutants include heavy metals (*28*–*30*) from point sources (i.e., hatting industry) and atmospheric emissions before environmental regulations came into place including the Clean Air Act (1963 and 1970) and Clean Water Act (1972). Population growth led to massive sewage input to the sound, which brought increased nutrients (phosphorus and nitrogen) and fresh water (*27*, *31*). The nutrient input led to eutrophication, plankton blooms, and seasonal deoxygenation of western LIS from the mid-19th century on (*10*, *32*), similar to that in other coastal waters (*33*, *34*). Pollution and deoxygenation are most concentrated in the western end of LIS, not only because of the location of major sources (New York City) but also due to the net transport of fine particulates enriched in pollutants from east to west (*28*, *29*, *35*, *36*).

Eutrophication-fueled plankton blooms in LIS were exacerbated by input of terrigenous organic matter (*31*, *32*) and overfishing of planktivores [oysters (*37*) and menhaden (*38*)]. Severe eutrophication may have affected phytoplankton communities through changes in the ratios of dissolved N, P, and Si, possibly resulting in the decline of Si-using (diatoms) relative to non-Si using phytoplankton [e.g., dinoflagellates; (*32*)]. Ecosystems were further affected by invasive species, including the European green crab [*Carcinus maenas*; 1784 to 1816; (*4*)] and the periwinkle [*Littorina littorea*; 1840 to 1890; (*4*)]. Along the coastline, the invasive reed *Phragmites australis* outcompeted the native *Phragmites americanus* (*39*). Benthic communities responded to these environmental changes (*40*, *41*); there is evidence of widespread community change in benthic foraminifera between the early 1960s and the mid-1990s, especially in western LIS (*40*, *42*–*45*), expressed as a major increase in relative and absolute abundance of taxa of the genus *Ammonia*, whose species-level taxonomy was not well understood until genetic analysis (*46*).

Here, we examine grab and core samples from LIS to investigate and document the history of *A. confertitesta*, a species identified as invasive (*46*), to make inferences about the time and mode of its introduction and reason(s) for its successful establishment and proliferation (*7*, *8*). Foraminifera are among the many small marine species commonly overlooked in compilations of invasive taxa (*14*). We propose that these often overlooked, unicellular, shelled eukaryotes may be used to document the relative timing of invasions and degradation in coastal regions (*4*, *7*), thereby adding materially to our understanding of the relative importance of species invasions in the degradation and management of ecosystems (*6*).

## RESULTS

**Fig. 1.**
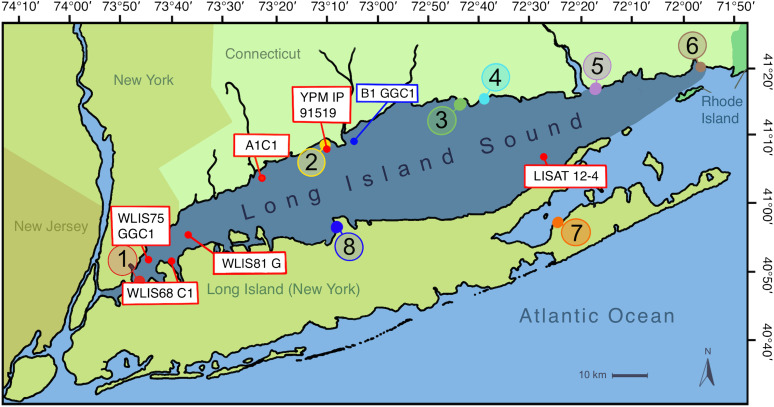
Map of Long Island Sound with sample sites. Sample locations for this study (red boxes) and one sample site from Fig. 4 (blue box), on a map showing sample locations from (*46*) (circles).

**Fig. 2.**
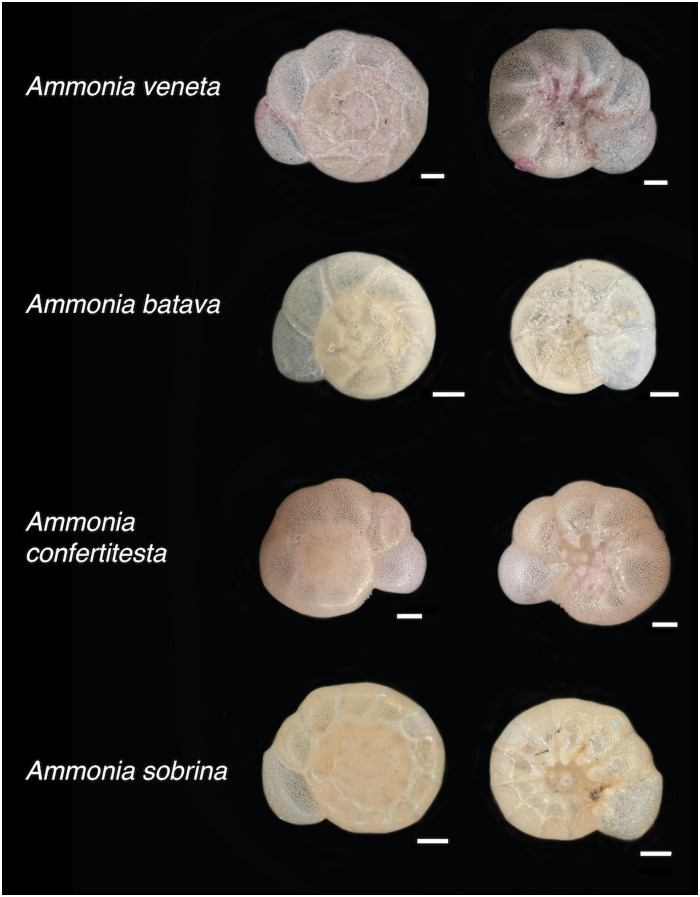
Representative individuals for each *Ammonia* species identified. Scale bars, 50 μm.

Across the five cores and museum samples (Buzas’ collection) spanning ~10,000 years and nearly the entire length of LIS (Fig. 1), we found four morphospecies: *Ammonia confertitesta, Ammonia veneta, Ammonia sobrina*, and *Ammonia batava* (Fig. 2). Four tests were identified as “likely *Ammonia sobrina*” in the oldest core samples (core LISAT 12-4, ~10,000 years BP), as well as one test from the early 16th century (core A1C1). We identified rare *A. batava* in core A1C1 (“likely *A. batava*,” approximately 1350 CE; Fig. 2) and in Buzas’ collection (*42*). *A. batava* is a species found along east and west Atlantic coasts (*47*) but not in modern LIS samples (*46*). The cosmopolitan *A. veneta* (*47*) was identified as living in LIS by Goetz *et al.* (*46*) as rare, and here, it was identified only in a Buzas sample from the 1960s (YPM IP 91519).

**Fig. 3.**
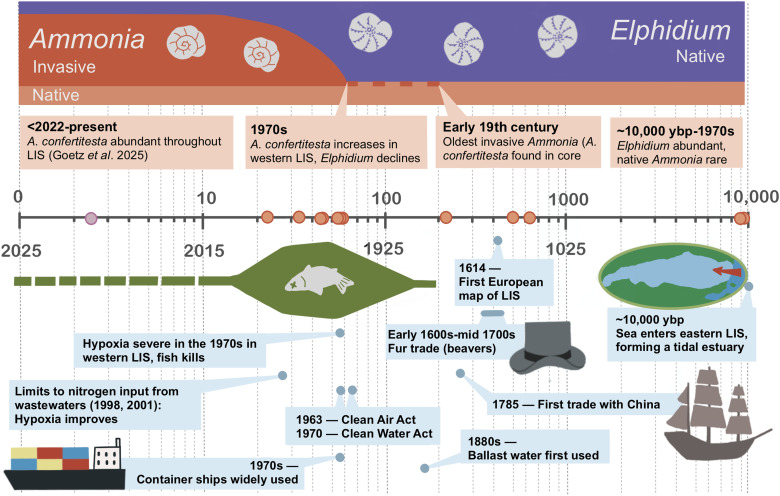
An ecological and environmental history of Long Island Sound. Timeline of *Ammonia* and *Elphidium* abundance (top) and important events in the ecological and environmental histories of LIS (bottom). Dots on the timeline represent core samples from this study (orange) and samples from Goetz *et al.* (*46*) (purple). ybp, years BP.

The oldest, rare, *A. confertitesta* (invasive species) in our samples was found in a sample from the early to mid-19th century (approximately 1819, core WLIS75 GGC1, westernmost LIS), and there were a few specimens in cores A1C1 and WLIS68-C1 in samples dated to 1961. We cannot absolutely prove the absence of *A. confertitesta* before the early 1800s because *Ammonia* specimens are so rare in samples from before the 1970s. Many more specimens of *A. confertitesta* were present in samples from approximately 1975 onward (cores WLIS68-C1 and WLI75GGC1 and grab sample WLIS81-G). For example, of the 30 specimens picked from WLIS81-G (2001), 23 were identified as *A. confertitesta* and the others were not identifiable. Similarly, all 12 identified specimens in WLIS 68-C1 (from 1978) were identified as *A. confertitesta*. We concluded that the increase in abundance of *Ammonia* species in general resulted from the increased abundance of the species *A. confertitesta*, which continued to spread eastward in LIS from the 1980s on (Fig. 3) (*32*, *40*, *45*, *46*).

## DISCUSSION

**Fig. 4.**
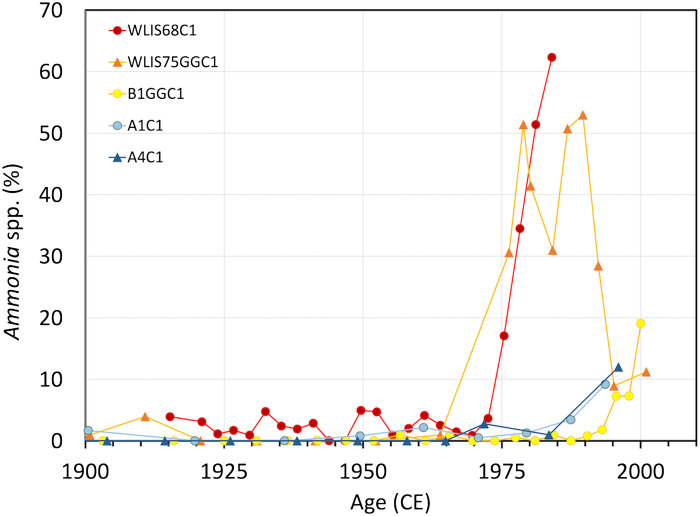
Relative abundance of *Ammonia* through time. Relative abundance of *Ammonia* (not identified to species level) in LIS cores; cores in red/orange colors further to the west, cores in blue further east [data after Varekamp *et al.* and Thomas *et al*. (*32*, *45*)].

**Fig. 5.**
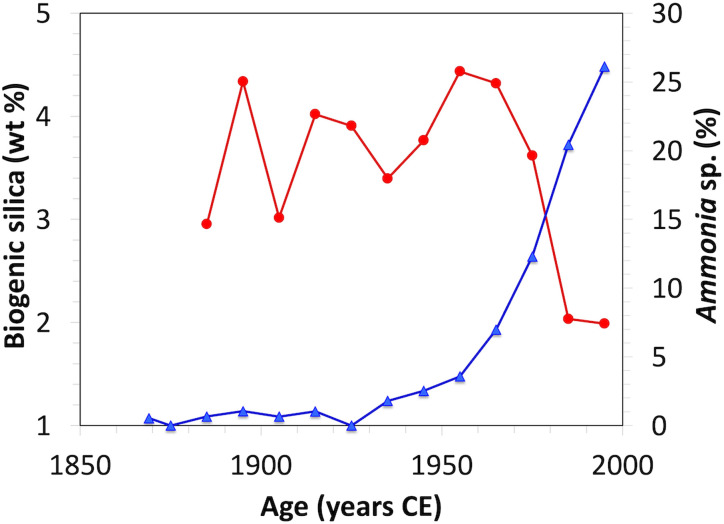
Biogenic silica (wt %) and *Ammonia* abundance through time. Wt % of biogenic silica (red) plotted with the percentage of *Ammonia* (blue) abundance; data in Varekamp *et al.* (*82*).

A pervasive change in LIS ecosystems is indicated by the qualitative shift in benthic foraminiferal communities in the mid-1970s (Figs. 4 and 5), with a major increase in specimens belonging to the genus *Ammonia* relative to the native *Elphidium excavatum*, dominant since the post–glacial establishment of LIS as an estuary (*40*, *42*–*45*, *48*). This increase in *Ammonia* started long after the initiation of environmental degradation in LIS in the mid-1800s (*32*, *45*) but was, despite the lack of agreement in timing, speculatively attributed to environmental factors such as eutrophication, deoxygenation, or temperature increase, although the mechanism(s) and/or driver(s) were never clear (*40*, *43*–*45*). Our results show that this major community change was driven by the proliferation of the non-native *A. confertitesta*—not to the sudden success of a native species.

The rare specimens of *Ammonia* species identified in core and museum samples older than the 1970s differed starkly from those sampled living presently (*46*). Among the rare *Ammonia* in older samples, we identified *A. sobrina*, *A. veneta*, *A. batava*, and *A. confertitesta*. The first three taxa are well documented along the eastern US seaboard (*47*). *A. sobrina* was first described morphologically from New York Harbor (*48*, *49*) and known genetically from Peconic Bay [eastern Long Island; (*50*)] and morphologically along both US coasts (*47*). The LIS native species *A. batava* (figured by Buzas in 1965; U.S.N.M.64126; Pl. 4, figs. 1a, 1b; assigned to *Ammonia beccarii*) was not seen in surface samples by Goetz *et al.* (*46*), but due to its low abundance in all samples where it is present (table S1), we do not know whether it is extant (although rare) in LIS. The native *A. sobrina* and cosmopolitan *A. veneta* remain rare in LIS today (*46*).

In contrast, the species *A. confertitesta* dominated communities after the mid-1970s, i.e., from the time of the sudden increase in abundance of the genus *Ammonia* (Fig. 3). The conclusion that the increase in the genus *Ammonia* is caused by the proliferation of the invasive *A. confertitesta*, despite the presence of specimens that cannot be identified with certainty (table S1), is strongly supported by the fact that extant *Ammonia* assemblages in LIS are likewise dominated by *A. confertitesta* (*46*). This species is thought to be native to East Asia [Jiaozhou Bay, China; (*51*)] and is reported as invasive in western Europe (*52*–*60*), where it appeared in the mid-1980s, i.e. shortly after its spread in LIS (*58*, *59*), and along the Pacific coast of Canada, where it arrived after approximately 2000 (*14*). *A. confertitesta* is now widespread and common in LIS; thus, it is highly likely that it can be found at nearby locations along the northeast coast of the US.

The oldest *A. confertitesta* identified with confidence was from a core in westernmost LIS (WLIS75GGC1, close to Execution Rock Lighthouse), in a sample dated at approximately 1820 CE. Invasive species commonly have a lag phase between their initial introduction and their rapid expansion, due to factors such as propagule pressure and species/environmental fit, as well as the status of the environments/ecosystems in which the species is introduced (*8*, *17*, *19*). Species may also have one or multiple failed introductions (i.e., followed by extirpations) before successfully taking hold. This pattern of successive failed introductions or lag followed by rapid increase in abundance is consistent with the hypothesis that *A. confertitesta* is non-native in LIS (*46*).

Was the increased abundance and ongoing eastward spread of *A. confertitesta* in LIS, after a lag period of ~150 years, caused by changes in the introduction process (i.e., a change in the dispersal vector), by environmental changes in LIS (i.e., a change in the recipient environment), or by some combination of the two?

*A. confertitesta* was most likely introduced through shipping (*14*, *52*). The earliest ship traffic between New York Harbor, from where migration through the East River into LIS would be possible, and China, where *A. confertitesta* is native (*47*), occurred in 1785 (*61*). Before the 1880s, ships did not use ballast water, but biofouling or ballast rocks may have been responsible for the initial introduction of *A. confertitesta* (*4*). In addition, the East River was widened considerably in the 1880s by heavy explosions, making migration easier (*62*).

The final success of *A. confertitesta* in LIS in the 1970s might well be due to an increase in propagule pressure from a rise in shipping traffic in New York Harbor, as well as a major increase in the number of ships carrying large amounts of ballast waters (containers and tankers) (*4*, *14*, *21*). The global movement of goods tripled since the early 19th century (*63*) and the volume of ballast water transported has increased since its first widespread use in the late 1900s to roughly 10 billion metric tons (10 km^3^) discharged every year in international trade in modern times (*64*). The hypothesis of increased propagule pressure due to container-ship ballast water transport is supported by the observation that the species appeared as an invasive in France and the UK in the mid-1980s, i.e., shortly after its development into an invasive in LIS (*56*, *58*, *59*, *65*). Its migration into western Europe thus might have been via the US East Coast. It was observed in about 2000 in Kiel Fjord and the Baltic Sea (*53*, *57*, *66*).

Environmental changes in LIS might have contributed to this success. Anthropogenic environmental changes in western LIS, where *A. confertitesta* would have arrived from New York, include metal pollution, declining salinity (*67*, *68*), eutrophication, and seasonal deoxygenation, all of which could have made successful invasion easier through negative effects on native faunas and ecosystem destabilization. There is, however, no evidence for a temporal correlation between these environmental changes and the successful invasion by *A. confertitesta* in the 1970s: All these factors show environmental deterioration in western LIS environments starting in the mid-1800s, as indicated by multiple microfossil and geochemical proxies (*29*, *32*, *45*).

Relative trends, timing, and geography of hypothesized environmental drivers (i.e., deoxygenation, phytoplankton community structure, and warming) provide further context for this interpretation. A high abundance of *Ammonia* spp. relative to *Elphidium* spp.—expressed as the A-E index (the ratio of *Ammonia* to *Elphidium*)—was proposed as a deoxygenation proxy in the Gulf of Mexico [e.g., (*69*, *70*)], despite laboratory observations that both *Ammonia* and *Elphidium* species are tolerant to low oxygen (*71*). In LIS surface samples, a west-east gradient in deoxygenation was correlated with a gradient in A-E index values (*43*, *44*). However, LIS oxygenation decreased significantly starting in the mid-1800s (*32*, *45*), long before the increase in the ratio of *Ammonia* to *Elphidium* (Fig. 5). Oxygenation may have started to improve gradually since the introduction of limits on nitrogen inputs from wastewater treatment plants in New York and Connecticut in 2001 by the US Environmental Protection Agency (EPA), with evidence for improving oxygenation from about 2006 to 2008 (*72*). However, surface sampling in 2023 documented that *A. confertitesta* was the dominant *Ammonia* as far east as the mouth of Connecticut River, where no seasonal deoxygenation occurs (*46*). Therefore, we see the observed correlation between a high A-E index and low oxygen conditions in surface samples in LIS (*45*) as indicative of correlation, not causation. We conclude that the high abundance of *A. confertitesta* in western LIS, where seasonal deoxygenation is more severe than further east, was primarily caused by its successful invasion, not by local environmental conditions. This insight could be provided only by genetic research at the species level (*46*). We conclude that changes in the A-E index are not a reliable proxy of oxygenation without information about the species driving the dynamics.

Another environmental factor, speculated to have caused increased abundance of *Ammonia* spp. relative to *Elphidium* spp. are changes in phytoplankton communities in LIS due to severe eutrophication (*40*, *45*). *Ammonia* and *Elphidium* differ in their diet, suggested as an additional control on the A-E index (*73*, *74*). Specifically, *Elphidium* depends on diatom-derived kleptochloroplasts (*75*–*77*), whereas *A. confertitesta* is omnivorous, feeding on algal material (*78*) and metazoans (*79*, *80*). It thus was suggested that the main cause of the increased A-E index in western LIS was a change in phytoplankton community (*40*, *45*), with decreased diatoms due to increasing N/Si, thereby giving an advantage to *Ammonia* [see also (*81*)]. At least in one core taken in westernmost LIS (WLIS 75-C1), the concentration of biogenic silica (produced by diatoms) declined at the same time as the increase in *Ammonia* species (*82*); thus, we cannot fully exclude this speculation, but further research is needed.

Global warming could be another potential factor contributing to the success of *A. confertitesta* since the mid-1970s. Some species of *Ammonia* are confined to tropical-subtropical zones (*47*) with optimal growth and reproductive temperatures between 20° and 30°C (*83*, *84*), but *A. confertitesta* occurs in generally more temperate conditions (*47*). In addition, LIS temperatures are fairly stable from west to east, and a high abundance of *A. confertitesta* is not linked to local LIS temperature, so it is unlikely that warming is a main reason for its success. In contrast, the recent appearance of the tropical *Ammonia advena* (T7) in LIS [(*46*); i.e., present in surface samples but not seen in historic samples] may be due to warming and northward range expansion.

In LIS, species in the foraminiferal genus *Ammonia* were rare from its establishment after the Last Ice Age until approximately 1970s. Our data indicate that the increase in its generic abundance, and in its abundance relative to that of *Elphidium* spp., was caused by the invasion of the Asian species *A. confertitesta.* The oldest observed *A. confertitesta* occurred in westernmost LIS in the early 19th century, suggesting arrival from New York Harbor ship traffic, possibly through biofouling. The species remained rare for about 150 years but became dominant in westernmost LIS in the mid-1970s and then migrated eastward. We hypothesize that the lag from the introduction of *A. confertitesta* to its spread as an invasive species was due to low propagule pressure: A massive increase in global shipping and ballast water volumes in the past 50 years coincided with the success of *A. confertitesta* as an invader in both the eastern US and western Europe. We cannot fully exclude the possibility that the success of this invasion was in part due to, or facilitated by, environmental degradation in LIS destabilizing the ecosystem. Core records of various environmental proxies do not support a significant correlation between the success of *A. confertitesta* and deoxygenation or increasing temperatures, but the potential effects of phytoplankton changes need more investigation. We thus show that foraminifera, because of their small size, abundance, and the presence of a calcite test (preservation potential), can be used to document the timing of introduction as well as the proliferation into a successful invader of aquatic species, even in the absence of contemporary observations.

## MATERIALS AND METHODS

### Experimental design

To document the timing of invasion in LIS, we examined *Ammonia* from collections and cores over an age range of several centuries and millennia and morphologically identified each to the species level, following Goetz *et al.* (*46*). Two of the specimens collected by Buzas in 1961 [described in (*42*)] were available in the Yale Peabody Museum collections (accession: YPM IP 91519, 259661), and we obtained a scanning electron microscopy (SEM) picture of the specimen figured by Buzas (1965) (U.S.N.M.64126; Pl. 4, figs. 1a, 1b; fig. S1) from the National Museum of Natural History (NMNH). In addition, we used grab and core samples collected by the USGS in 1984 and 1996/1997 and Wesleyan University in 2001 to consider the relative abundance and identity of *Ammonia*. Core samples were dated by ^210^Pb/ ^137^Cs, ^14^C, and the signature of mercury pollution (see fig. S1 and table S1).

We examined all *Ammonia* specimens from Buzas’s samples from the 1960s that were preserved in collections at Yale and the NMNH. *Ammonia* specimens were imaged using a Keyence VHX-7000 light microscope. Images included the spiral and umbilical sides of each individual at 500× magnification, unless cells were unusually small or large, in which case they were imaged at 700× or 400×, respectively. When possible, the pores of the spiral side of the penultimate chamber were imaged at 1500×. Images were deposited in Zenodo (DOI: 10.5281/zenodo.15232945); see table S1 for metadata.

We assigned specimens to morphotypes on the basis of the study by Hayward *et al.* (*47*), Richirt *et al.* (*55*, *56*), and Pavard *et al.* (*60*), following the methods and discernible features described by Goetz *et al.* (*46*) for *A. veneta*, *A. confertitesta*, *A. advena*, and *A. sobrina.* For *A. batava,* which was not found by Goetz *et al.* (*46*), we referred primarily to Hayward *et al.* (*47*) and found that the most distinguishing features for our samples were their broad, smooth, partially open sutures and pronounced boss and beads on the umbilical side. Specimens were labeled by morphotype if we were confident in our assessment, by “likely” with the morphotype name if they had some, but not all identifying features, and by “unknown” if we were unable to assign a taxon name. For “unknown” individuals, we also note the reason for the impossibility of morphotype assignment, including “dissolution” for decalcified tests, “deformed” for tests with obvious deformities, “too few chambers,” and “ambiguous morph” for ambiguous morphologies (fig. S2). We found decalcified specimens in samples as old as approximately 1355 CE but none that were unidentifiable due to dissolution until the 1960s (fig. S3). Tests with deformities were found in samples ranging in age from ~10,000 years old through 2001.

## Supplementary Materials

### This PDF file includes:

Supplementary Text Figs. S1 to S3 Table S1 References
