# Supplementary material for: Foraminifera as indicators of species invasion: Ammonia confertitesta in Long Island Sound
Source: Sci Adv. 2025 Sep 3;11(36):eadv9447. doi: 10.1126/sciadv.adv9447 (PMC12407076; doi:10.1126/sciadv.adv9447)
Supplement: Supplementary file 1 — Supplementary Text Figs. S1 to S3 Table S1 References [file sciadv.adv9447_sm.pdf]

Science Advances
AAAS

Supplementary Materials for

Foraminifera as indicators of species invasion: *Ammonia confertitesta* in Long Island Sound

Eleanor J. Goetz *et al.*

Corresponding author: Eleanor J. Goetz, elly.goetz@yale.edu

Sci. Adv. **11**, eadv9447 (2025)
DOI: 10.1126/sciadv.adv9447

This PDF file includes:

Supplementary Text
Figs. S1 to S3
Table S1
References

Supplementary Text

Cores

Core LISAT 12: In 1984, R.S. Lewis and co-workers collected 13 vibracores in LIS (MMS-cores; 85) using the R/V Atlantic Twin. Core LISAT12 was obtained near the Long Island coast to the southwest of the mouth of the Connecticut River (41°07.70'N, 72°28.80'W; point 25, map 2784, (86) at a water depth of 37 m. All core data (including the cruise report, visual core descriptions, core photographs) are available online (93). We took 40 samples from core LISAT12 (curated at the Woods Hole Oceanographic Institution) and submitted material from 8 depth intervals for radiocarbon dating at NOSAMS, WHOI. We submitted mollusk (carbonate) shell fragments from all 8 samples, and from 5 samples we also submitted hand-picked macrofloral remains, small twigs and leaf fragments, which are abundant in these sediments. Data were published in (87-89). The samples used to obtain *Ammonia* spp. for this study all contained oysters, and foraminiferal assemblages are dominated by *Elphidium* spp. (89).

Core A1C1, Grab sample WLIS81-G, Core WLIS 68C1, Core WLIS 75C1. The US Geological Survey collected sediment surface and core samples on two cruises in 1996 (R/V Seaward Explorer: SEAX96017, SEAX96024), and one cruise in 1997 in western Long Island Sound the (R/V John Dempsey, JD97001). Surface sediment was collected with Van Veen grab sampler with video and still camera. Sediment cores (between 1 and 6 replicates per location) were taken using the U.S. Geological Survey's hydrostatically- damped gravity corer (90) which collected 11-cm diameter cores up to 70 cm in length in clear, polycarbonate tubing. For materials collected in 1996, stations received a station identification consisting of a letter and a number (e.g., A1); stations on transects at right angles to the Connecticut shoreline received the same letter (e.g., stations for cores A1 and A4 are on one transect). Cores were labeled with the Station ID followed by C (for core), followed by a number for the replicate (e.g., A1C1 is the first core taken at Station A1). Grab samples were labeled by the station number followed by the letter G: A1G1 is a grab sample taken at the same location as core A1C1. Materials collected in 1997 have station IDs of the letters WLIS followed by a number: WLIS81-G is a grab sample taken at station 81 in the western Long Island Sound. Information on core and grab samples is provided by (*27–29*, *43*, *44*,, 82, 91), ; and is available online (92, 93).

Core WLIS75-GGC1: this core was collected in October 2001 on a joint USGS-Wesleyan University cruise with the R/V Connecticut (CONN01066), on which 14 long cores (175-243 cm) were collected at stations first sampled by the USGS in 1996-1997, and given the station number followed by the letters GGC to indicate a long core, and a number indicating replicate cores (32, 94, 95).

Section 2: Age models

Radiocarbon dates were collected in cores A1C1 and WLIS75GGC1 on carbonate shells rather than on organic matter, because organic carbon ages tend to be too old, i.e., they reflect the time that the plant died, which is not necessarily the time of deposition of the mud layer in which it is now contained. Ages derived from carbonate secreting organisms that are living *in situ* approach the age of deposition much better, despite issues of radiocarbon reservoir effects (89). The upper part of core A1C1 was analyzed for ^{210}Pb and ^{137}Cs, the first a naturally occurring radioisotope with a dating time span of about 150 years. The isotope ^{137}Cs derives from atmospheric nuclear tests, which started in the early 1950s and peaked in 1964 (*35*, *87*). Bioturbation blurs the ^{137}Cs signal, making the resolution less than the five to ten years that is theoretically possible, although we concentrated our efforts on cores with minimum bioturbation, as determined from core X-rays. We used chemostratigraphy, i.e., the record of anthropogenic mercury (Hg) pollution levels dated on land and by the historical record, for additional age control (*35*).

Fig. S1.

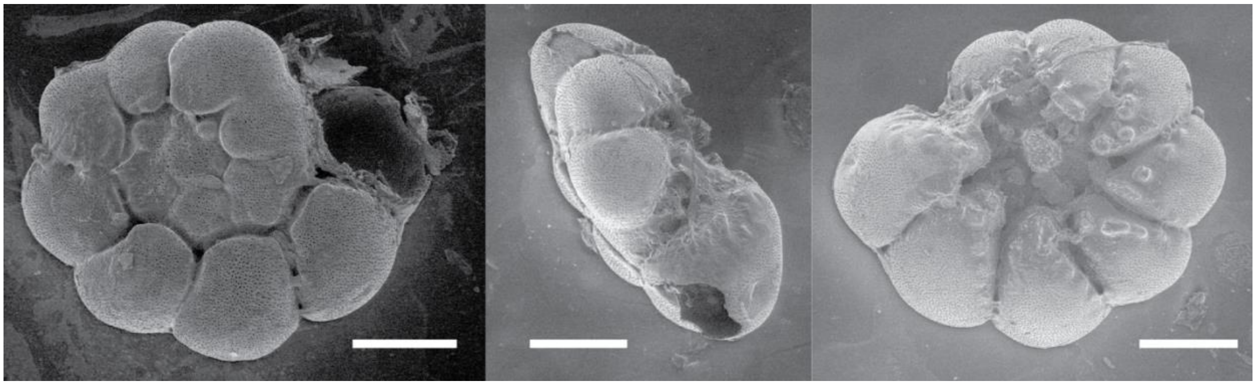

SEM image of *Ammonia batava.* Specimen figured by Buzas (1965) (U.S.N.M.64126; Pl. 4, figs1a, 1b; (*42*); Image courtesy of Brian Huber, Smithsonian National Museum of Natural History

Fig. S2.

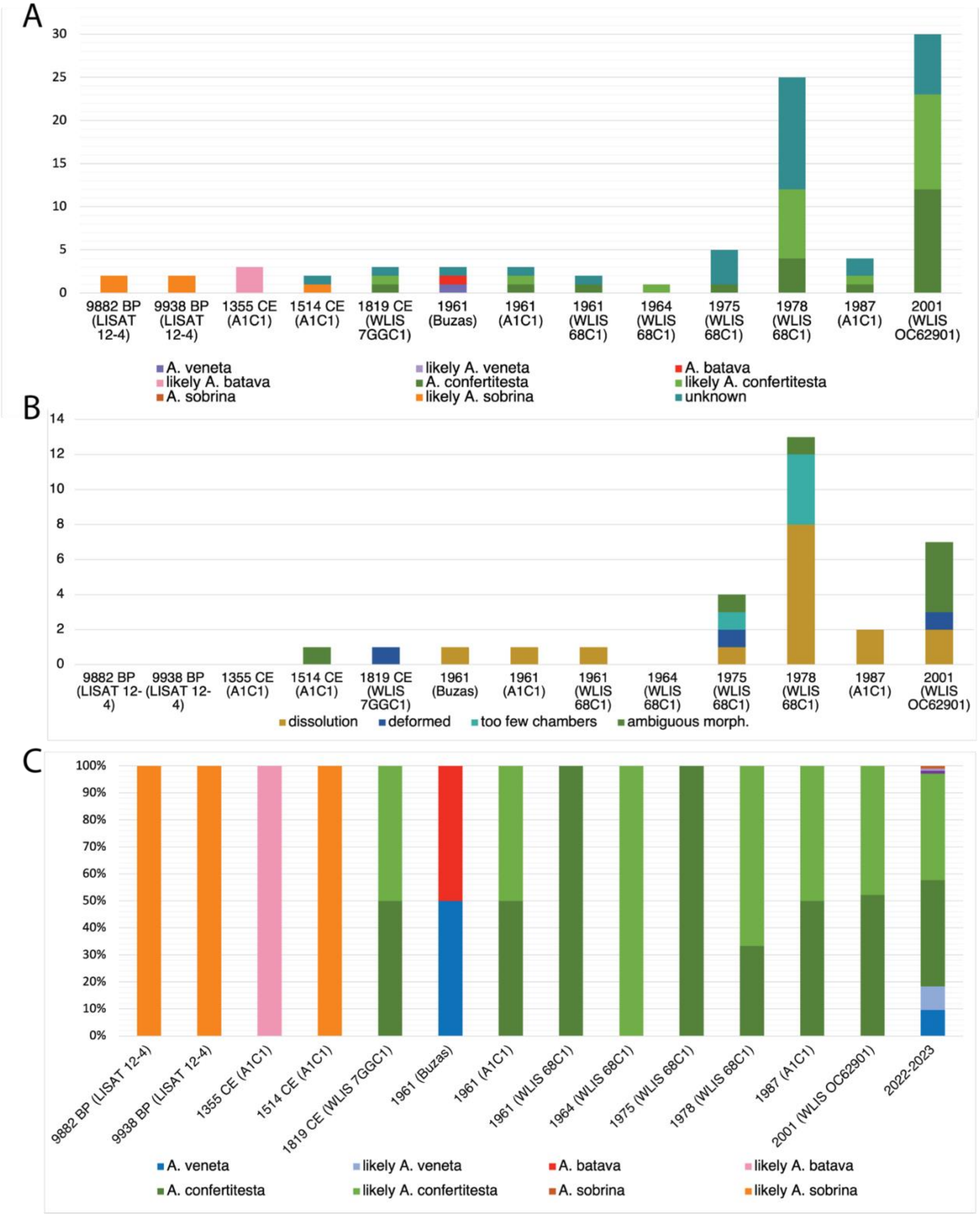

Breakdown of morphological identifications by site. (A) Number of specimens for each morphological identity (B) Breakdown of specimens labeled as unknown (i.e., too dissolved, too deformed, too few chambers, too ambiguous to be identified) (C) Percentage of each morphological identity in each sample, including modern samples of living *Ammonia* from Goetz et al. (2025; *45*)

Fig. S3.

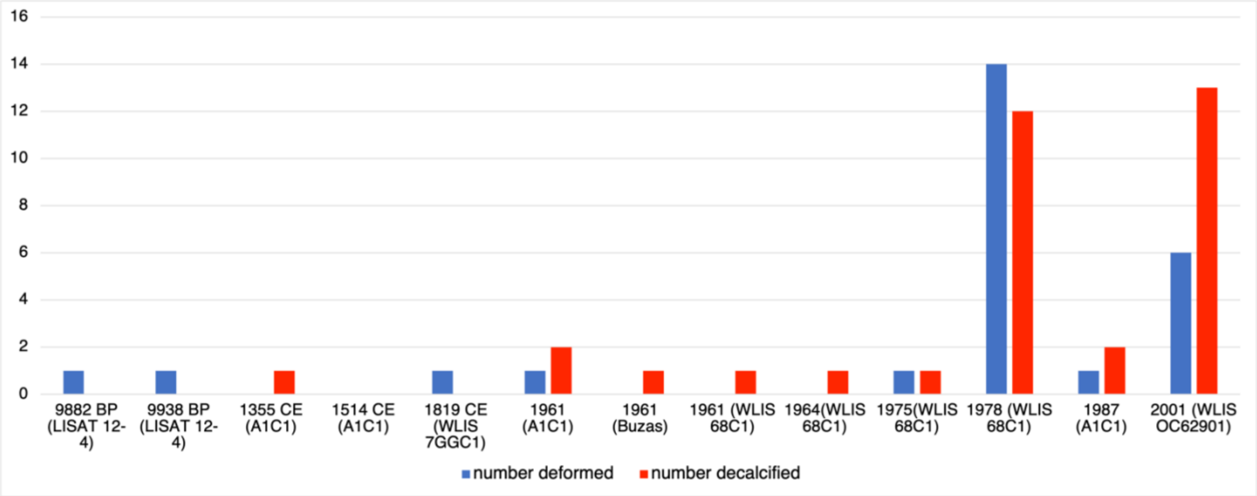

The number of deformed and decalcified tests for each site (i.e., the tests that are deformed or decalcified in any way, not necessarily too deformed or decalcified to be identified).

Table S1.
Metadata for sites, samples, and specimens.

**Core or Museum ID**	**Coordinates**	**Depth-in-core (cm)**	**Approximate age**	**# of tests imaged**	**taxa identified**
LISAT 12-4 (core)	(41.128334, -72.479996)	26-28 (521)	9880 yr BP	2	Likely *A. sobrina* (2)
LISAT 12-4 (core)	(41.128334, -72.479996)	44-46 (539)	9940 yr BP	2	Likely *A. sobrina* (2)
A1C1 (core)	(41.09888, -73.33173)	47.5-50	1355 CE	3	Likely *A. batava* (3)
A1C1 (core)	(41.09888, -73.33173)	42.5-43.0	1514 CE	2	Likely *A. sobrina* (1); Unknown (ambiguous morphology) (1)
A1C1 (core)	(41.09888, -73.33173)	10-10.5	1961 CE	3	*A. confertitesta* (1); Likely *A. confertitesta* (1); Unknown (dissolution) (1)
A1C1 (core)	(41.09888, -73.33173)	2.5-3.0	1987 CE	4	*A. confertitesta* (1); Likely *A. confertitesta* (1); Unknown (dissolution –2)
WLIS75GGC1 (core; 2001)	(40.87792, -73.74622)	79-80	1819 CE	3	*A. confertitesta* (1); Likely *A. confertitesta* (1); Unknown (deformed test – 1)
WLIS81G (grab; 2001)	(40.93122, 73.63132)	Surface	2001 CE	30	*A. confertitesta* (12); Likely *A. confertitesta* (11); Unknown (deformed test –1; dissolution – 2; ambiguous morphology – 4)
WLIS 68C1 (core)	(40.86602, -73.66458)	26-28	1961 CE	2	*A. confertitesta* (1); Unknown (dissolution – 1)
WLIS 68C1 (core)	(40.86602, -73.66458)	24-26	1964 CE	1	Likely *A. confertitesta* (1)
WLIS 68C1 (core)	(40.86602, -73.66458)	16-18	1975 CE	5	*A. confertitesta* (1); Unknown (dissolution – 1; deformed –1; too few chambers– 1; ambiguous morphology – 1)
WLIS 68C1 (core)	(40.86602, -73.66458)	14-16	1978 CE	25	*A. confertitesta* (4); Likely *A. confertitesta* (8); (too few chambers –4; dissolution – 8; ambiguous morphology – 1)

Peabody # YPM IP 91519 (Buzas) (grab; 9161)	~ (41.15098, -73.18763)	Surface	1961 CE	1	*A. veneta* (1)
Peabody # YPM IP 259661(grab; 9161)	~ (41.15098, -73.18763)	Surface	1961 CE	1	Unknown (dissolution – 1)
U.S.N.M.64126 (Buzas) (grab; 9161)	~ (41.15098, -73.18763)	Surface	1961 CE	1	*A. batava* (1)
			total	84	

REFERENCES AND NOTES

1. N. C. James, T. D. Leslie, W. M. Potts, A. K. Whitfield, A. Rajkaran, The importance of different juvenile habitats as nursery areas for a ubiquitous estuarine-dependent marine fish species. *Estuar. Coast. Shelf Sci.* **226**, 106270 (2019).

2. S. Booi, S. Mishi, O. Andersen, Ecosystem services: A systematic review of provisioning and cultural ecosystem services in estuaries. *Sustainability* **14**, 7252 (2022).

3. M. J. Kennish, Environmental threats and environmental future of estuaries. *Environ. Conserv.* **29**, 78–107 (2002).

4. H. Ojaveer, B. S. Galil, J. T. Carlton, H. Alleway, P. Goulletquer, M. Lehtiniemi, A. Marchini, W. Miller, A. Occhipinti-Ambrogi, M. Peharda, G. M. Ruiz, S. L. Williams, A. Zaiko, Historical baselines in marine bioinvasions: Implications for policy and management. *PLOS ONE* **13**, e0202383 (2018).

5. S. L. Williams, E. D. Grosholz, The invasive species challenge in estuarine and coastal environments: Marrying management and science. *Estuaries Coasts* **31**, 3–20 (2008).

6. J. T. Carlton, E. Schwindt, The assessment of marine bioinvasion diversity and history. *Biol. Invasions* **26**, 237–298 (2024).

7. G. J. Vermeij, An agenda for invasion biology. *Biol. Conserv.* **78**, 3–9 (1996).

8. J. T. Carlton, Pattern, process, and prediction in marine invasion ecology. *Biol. Conserv.* **78**, 97–106 (1996).

9. J. S. Latimer, M. A. Tedesco, R. L. Swanson, C. Yarish, P. E. Stacey, C. Garza, Eds. *Long Island Sound: Prospects for the Urban Sea*, Springer Series on Environmental Management (Springer New York, 2014).

10. C. A. Parker, J. E. O'Reilly, Oxygen depletion in Long Island Sound: A historical perspective. *Estuaries* **14**, 248 (1991).

11. M. Duvall, J. Hagy III, J. Ammerman, M. Tedesco, High-frequency dissolved oxygen dynamics in an urban estuary, the Long Island Sound. *Estuaries Coasts* **47**, 415–430 (2023).

12. S. Nicholls, *Paradise Found: Nature in America at the Time of Discovery* (University of Chicago Press, 2009).

13. M. McGann, G. M. Ruiz, A. H. Hines, G. Smith, A ship's ballasting history as an indicator of foraminiferal invasion potential—An example from Prince William Sound, Alaska, USA. *J. Foram. Res.* **49**, 434–455 (2019).

14. M. McGann, M. Holzmann, First occurrence of the nonindigenous Asian foraminifera Ammonia confertitesta in the Northeastern Pacific Ocean: Vancouver Island, British Columbia, Canada. *Micropaleontology* **70**, 115–127 (2024).

15. A. Lacoursière-Roussel, D. G. Bock, M. E. Cristescu, F. Guichard, C. W. McKindsey, Effect of shipping traffic on biofouling invasion success at population and community levels. *Biol. Invasions* **18**, 3681–3695 (2016).

16. M. Williamson, A. Fitter, The varying success of invaders. *Ecology* **77**, 1661–1666 (1996).

17. J. A. Crooks, M. E. Soulé, "Lag times in population explosions of invasive species: Causes and implications" in *Invasive Species and Biodiversity Management*, O. T. Sandlund, P. J. Schei, Å. Viken, Eds. (Springer Netherlands, 1999), pp. 103–125.

18. J. L. Lockwood, P. Cassey, T. Blackburn, The role of propagule pressure in explaining species invasions. *Trends Ecol. Evol.* **20**, 223–228 (2005).

19. D. Simberloff, The role of propagule pressure in biological invasions. *Annu. Rev. Ecol. Evol. Syst.* **40**, 81–102 (2009).

20. N. Bax, A. Williamson, M. Aguero, E. Gonzalez, W. Geeves, Marine invasive alien species: A threat to global biodiversity. *Mar. Policy* **27**, 313–323 (2003).

21. I. C. Davidson, C. Scianni, M. S. Minton, G. M. Ruiz, A history of ship specialization and consequences for marine invasions, management and policy. *J. Appl. Ecol.* **55**, 1799–1811 (2018).

22. P. J. Prentis, J. R. U. Wilson, E. E. Dormontt, D. M. Richardson, A. J. Lowe, Adaptive evolution in invasive species. *Trends Plant Sci.* **13**, 288–294 (2008).

23. J. S. Weis, Invasion and predation in aquatic ecosystems. *Curr. Zool.* **57**, 613–624 (2011).

24. P. R. Ehrlich, "Which animal will invade?" in *Ecology of Biological Invasions of North America and Hawaii*, H. A. Mooney, J. A. Drake, Eds. (Springer New York, 1986), pp. 79–95.

25. J. C. Varekamp, The historic fur trade and climate change. *Eos Trans. Am. Geophys. Union* **87**, 593–597 (2006).

26. B. Goldfarb, *Eager: The Surprising, Secret Life of Beavers and Why They Matter* (Chelsea Green Publishing, 2018).

27. M. R. Buchholtz ten Brink, E. L. Mecray, E. L. Galvin, Clostridium perfringens in Long Island Sound sediments: An urban sedimentary record. *J. Coast. Res.* **16**, 591–612 (2000).

28. E. L. Mecray, M. R. B. ten Brink, Contaminant distribution and accumulation in the surface sediments of Long Island Sound. *J. Coast. Res.* **16**, 575–590 (2000).

29. J. C. Varekamp, M. R. B. ten Brink, E. L. Mecray, B. Kreulen, Mercury in Long Island Sound sediments. *J. Coast. Res.* **16**, 613–626 (2000).

30. E. Thomas, J. Varekamp, "Sea Level Rise in Long Island Sound Over the Last Millennium," in *AGU Fall Meeting Abstracts* (American Geophysical Union, 2002).

31. P. Vlahos, M. M. Whitney, C. Menniti, J. R. Mullaney, J. Morrison, Y. Jia, Nitrogen budgets of the Long Island Sound estuary. *Estuar. Coast. Shelf Sci.* **232**, 106493 (2020).

32. J. C. Varekamp, E. Thomas, M. Altabet, S. Cooper, H. Brinkhuis, F. Sangiorgi, T. Donders, M. Buchholtz ten Brink, "Environmental Change in Long Island Sound in the Recent Past:

Eutrophication and Climate Change” (Grant #CWF 334-R, FRS #525156, Yale Univ., 2010), pp 1–54.

33. M. Dai, Y. Zhao, F. Chai, M. Chen, N. Chen, Y. Chen, D. Cheng, J. Gan, D. Guan, Y. Hong, J. Huang, Y. Lee, K. Leung, P. Lim, S. Lin, X. Lin, X. Liu, Z. Liu, Y. Luo, F. Meng, C. Sangmanee, Y. Shen, K. Uthaipan, W. I. A. W. Talaat, X. S. Wan, C. Wang, D. Wang, G. Wang, S. Wang, Y. Wang, Y. Wang, Z. Wang, Z. Wang, Y. Xu, J T. Yang, Y. Yang, M. Yasushara, D. Yu, L. Yu, Z. Zhang, Z. Zhang, Persistent eutrophication and hypoxia in the coastal ocean. *Camb. Prism. Coast. Futures* **1**, e19 (2023).

34. N. N. Rabalais, W.-J. Cai, J. Carstensen, D. J. Conley, B. Fry, X. Hu, Z. Quinones-Rivera, R. Rosenberg, C. P. Slomp, R. E. Turner, M. Voss, B. Wissel, J. Zhang, Eutrophication-driven deoxygenation in the coastal ocean. *Oceanography* **27**, 172–183 (2014).

35. J. Varekamp, B. Kreulen, B. M. Ten Brink, E. Mecray, Mercury contamination chronologies from Connecticut wetlands and Long Island Sound sediments. *Environ. Geol.* **43**, 268–282 (2003).

36. J. Varekamp, E. Mecray, T. Maccalous, “Once spilled, still found: Metal contamination in Connecticut coastal wetlands and Long Island Sound sediment from historic industries” in *America’s Changing Coasts: Private Rights And Public Trust* (Edward Elgar Publishing, 2005).

37. M. Kurlansky, *The Big Oyster: History on the Half Shell* (Random House, 2007).

38. H. B. Franklin, *The Most Important Fish in the Sea: Menhaden and America* (Island Press, 2007).

39. K. M. Kettenring, D. F. Whigham, E. L. G. Hazelton, S. K. Gallagher, H. M. Weiner, Biotic resistance, disturbance, and mode of colonization impact the invasion of a widespread, introduced wetland grass. *Ecol. Appl.* **25**, 466–480 (2015).

40. G. Lopez, D. Carey, J. Carlton, R. Cerrato, H. Dam, R. DiGiovanni, C. Elphick, M. Frisk, C. Gobler, L. Hice, P. Howell, A. Jordaan, S. Lin, S. Liu, D. Lonsdale, M. McEnroe, G. McManus, R. Orson, B. Peterson, C. Pickerell, R. Rozsa, S. Shumway, A. Siuda, K. Streich, S. Talmage, G.

Taylor, E. Thomas, M. Van Patten, J. Vaudrey, C. Yarish, G. Wikfors, R. Zajac, "Biology and ecology of Long Island Sound" in *Long Island Sound: Prospects for the Urban Sea* (Springer New York, 2014).

41. M. M. Casey, G. P. Dietl, D. M. Post, D. E. G. Briggs, The impact of eutrophication and commercial fishing on molluscan communities in Long Island Sound, USA. *Biol. Conserv.* **170**, 137–144 (2014).

42. M. A. Buzas, *The Distribution and Abundance of Foraminifera in Long Island Sound*, Smithsonian Institution Miscellaneous Collection (Smithsonian Institution, 1965), vol. **149**, pp. 1–88).

43. E. Thomas, T. Gapotchenko, J. C. Varekamp, E. I. Mecray, M. R. Buchholtz ten Brink, Benthic foraminifera and environmental changes in Long Island Sound. *J. Coast. Res.* **16**, 641–655 (2000).

44. E. Thomas, T. Gapotchenko, J. C. Varekamp, E. L. Mecray, M. R. Buchholtz ten Brink, "Maps of Benthic Foraminiferal Distribution and Environmental Changes in Long Island Sound between the 1940s and the 1990s" (USGS, 2000).

45. E. Thomas, I. Abramson, J. Varekamp, M. R. Brink, "Eutrophication of Long Island Sound as traced by benthic foraminifera," in *Proceedings of the 6th Biennial Long Island Sound Research Conference* (National Oceanic and Atmospheric Administration, 2002), pp. 87–91.

46. E. Goetz, A. Yan, P. Hull, E. Thomas, *Ammonia* (Foraminifera) in Long Island Sound: Molecular and morphological diversity. *J. Foram. Res.* **55**, 45–59 (2025).

47. B. W. Hayward, M. Holzmann, J. Pawlowski, J. Parker, T. Kaushikk, M. Toyofuku, M. Tsuchiya, Molecular and morphological taxonomy of living *Ammonia* and related taxa (Foraminifera) and their biogeography. *Micropaleontology* **67**, 109–274 (2021).

48. F. L. Parker, Foraminiferal distribution in the Long Island Sound-Buzzards Bay area. *Bull. Mus. Comp. Zool.* **106**, 391–473 (1952).

49. B. Shupack, Some foraminifera from western Long Island Sound and New York Harbor. *Am. Mus. Novit.* **737**, 12 (1934).

50. J. Pawlowski, I. Bolivar, J. Farhni, L. Zaninetti, DNA analysis of “Ammonia beccarii” morphotypes: One or more species? *Mar. Micropaleontol.* **26**, 171–178 (1995).

51. S.-Y. Zheng, T. C. Cheng, X. T. Wang, Z. X. Fu, The Quaternary foraminifera of the Dayuzhang irrigation area, Shandong Province, and a preliminary attempt at an interpretation of its depositioal environment. *Stud. Mar. Sin.* **13**, 16–78 (1978).

52. J. Pawlowski, M. Holzmann, Diversity and geographic distribution of benthic foraminifera: A molecular perspective. *Biodivers. Conserv.* **17**, 317–328 (2008).

53. M. Schweizer, I. Polovodova, A. Nikulina, J. Schönfeld, Molecular identification of *Ammonia* and *Elphidium* species (Foraminifera, Rotaliida) from the Kiel Fjord (SW Baltic Sea) with rDNA sequences. *Helgol. Mar. Res.* **65**, 1–10 (2011).

54. S. A. Saad, C. M. Wade, Seasonal and spatial variations of saltmarsh benthic foraminiferal communities from North Norfolk, England. *Microb. Ecol.* **73**, 539–555 (2017).

55. J. Richirt, M. Schweizer, V. M. P. Bouchet, A. Mouret, S. Quinchard, F. J. Jorissen, Morphological distinction of three *Ammonia* phylotypes occurring along European coasts. *J. Foram. Res.* **49**, 76–93 (2019).

56. J. Richirt, M. Schweizer, A. Mouret, S. Quinchard, S. A. Saad, V. M. P. Bouchet, C. M. Wade, F. J. Jorissen, Biogeographic distribution of three phylotypes (T1, T2 and T6) of *Ammonia* (foraminifera, Rhizaria) around Great Britain: New insights from combined molecular and morphological recognition. *J. Micropalaeontol.* **40**, 61–74 (2021).

57. C. Bird, M. Schweizer, A. Roberts, W. E. N. Austin, K. L. Knudsen, K. M. Evans, H. L. Filipsson, M. D. J. Sayer, E. Geslin, K. F. Darling, The genetic diversity, morphology, biogeography, and taxonomic designations of Ammonia (Foraminifera) in the Northeast Atlantic. *Mar. Micropaleontol.* **155**, 101726 (2020).

58. J.-C. Pavard, V. M. P. Bouchet, J. Richirt, A. Courleux, E. Armynot Du Châtelet, G. Duong, R. Abraham, J.-P. Pezy, J.-C. Dauvin, L. Seuront, Preferential presence in harbours confirms the non-indigenous species status of Ammonia confertitesta (Foraminifera) in the English Channel. *Aquat. Invasions* **18**, 351–369 (2023).

59. J.-C. Pavard, J. Richirt, L. Seuront, H. Blanchet, M. P. A. Fouet, S. Humbert, B. Gouillieux, G. Duong, V. M. P. Bouchet, The great shift: The non-indigenous species Ammonia confertitesta (Foraminifera, Rhizaria) outcompetes indigenous Ammonia species in the Gironde estuary (France). *Estuar. Coast. Shelf Sci.* **289**, 108378 (2023).

60. J.-C. Pavard, J. Richirt, L. Courcot, P. Bouchet, L. Seuront, V. M. P. Bouchet, Fast and reliable identification of *Ammonia* phylotypes T1, T2 and T6 using a stereomicroscope: Implication for large-scale ecological surveys and monitoring programs. *Water* **13**, 3563 (2021).

61. New York City WPA Writers' Project, *A Maritime History of New York* (Going Coastal, 2004).

62. Smithsonian's National Museum of American History, The William Steinway Diary, 1861-1896: Hell Gate Explosion (2025); www.americanhistory.si.edu/steinwaydiary/annotations/?id=2030.

63. P. Hulme, Unwelcome exchange: International trade as a direct and indirect driver of biological invasions worldwide. *One Earth* **4**, 666–679 (2021).

64. M. De Souza, "Ballast water" in *Unconventional Water Resources*, M. Qadir, V. Smakhtin, S. Koo-Oshima, E. Guenther, Eds. (Springer International Publishing, 2022), pp. 213–229.

65. J. Richirt, A. Guihéneuf, A. Mouret, M. Schweizer, C. P. Slomp, F. J. Jorissen, A historical record of benthic foraminifera in seasonally anoxic Lake Grevelingen, the Netherlands. *Palaeogeogr. Palaeoclimatol. Palaeoecol.* **599**, 111057 (2022).

66. I. Polovodova, A. Nikulina, J. Schönfeld, W.-C. Dullo, Recent benthic foraminifera in the Flensburg Fjord (Western Baltic Sea). *J. Micropalaeontol.* **28**, 131–142 (2009).

67. J. C. Varekamp, A. McElroy, J. Mullaney, V. Breslin, “Chapter 5: Metals, organic compounds, and nutrients in Long Island Sound: Sources, magnitudes, trends and impacts” in *Long Island Sound: Prospects for the Urban Sea*, Springer Series on Environmental Management (Springer, 2013), pp. 320–435.

68. J. O’Donnell, R. E. Wilson, K. Lwiza, W. F. Bohlen, D. Codiga, D. B. Fribance, T. Fake, M. Bowman, J. C. Varekamp, “Chapter 3: The physical oceanography of Long Island Sound” in *Long Island Sound: Prospects for the Urban Sea*, Springer Series on Environmental Management (Springer, 2013), pp. 132–242.

69. B. K. Sen Gupta, R. Eugene Turner, N. N. Rabalais, Seasonal oxygen depletion in continental-shelf waters of Louisiana: Historical record of benthic foraminifers. *Geology* **24**, 227 (1996).

70. B. K. S. Gupta, E. Platon, Tracking past sedimentary records of oxygen depletion in coastal waters: Use of the Ammonia-Elphidium foraminiferal index. *J. Coast. Res.*, **III**, 1351–1355 (2006).

71. L. Moodley, C. Hess, Tolerance of infaunal benthic foraminifera for low and high oxygen concentrations. *Biol. Bull.* **183**, 94–98 (1992).

72. Connecticut Department of Energy and Environmental Protection, Interstate Environmental Commission, US Environmental Protection Agency, Long Island Sound Office, “2021 Long Island Sound Hypoxia Season Review” (Connecticut Department of Energy and Environmental Protection, 2021); https://portal.ct.gov/-/media/deep/water/lis_water_quality/monitoring/2021/2021-combined-report_final.pdf?rev=033db2b95bda40179abfdc7a066b1a85&hash=8594D5D655E0B9A37106DEE0327563D9.

73. C. A. Brunner, Hypoxia hotspots in the Mississippi Bight. *J. Foram. Res.* **36**, 95–107 (2006).

74. A. J. Gooday, F. Jorissen, L. A. Levin, J. J. Middelburg, S. W. A. Naqvi, N. N. Rabalais, M. Scranton, J. Zhang, Historical records of coastal eutrophication-induced hypoxia. *Biogeosciences* **6**, 1707–1745 (2009).

75. J. M. Bernhard, S. S. Bowser, Benthic foraminifera of dysoxic sediments: Chloroplast sequestration and functional morphology. *Earth Sci. Rev.* **46**, 149–165 (1999).

76. M. Correia, J. Lee, Chloroplast retention by Elphidium excavatum (Terquem). Is it a selective process? *Symbiosis* **29**, 343–355 (2000).

77. N. Glock, Benthic foraminifera and gromiids from oxygen-depleted environments—Survival strategies, biogeochemistry and trophic interactions. *Biogeosciences* **20**, 3423–3447 (2023).

78. P.-Y. Pascal, C. Dupuy, P. Richard, N. Niquil, Bacterivory in the common foraminifer Ammonia tepida: Isotope tracer experiment and the controlling factors. *J. Exp. Mar. Biol. Ecol.* **359**, 55–61 (2008).

79. C. Dupuy, L. Rossignol, E. Geslin, P.-Y. Pascal, Predation of mudflat meio-macrofaunal metazoans by a calcareous foraminifer, Ammonia tepida (Cushman, 1926). *J. Foram. Res.* **40**, 305–312 (2010).

80. P.-M. Chronopoulou, I. Salonen, C. Bird, G.-J. Reichart, K. A. Koho, Metabarcoding insights into the trophic behavior and identity of intertidal benthic foraminifera. *Front. Microbiol.* **10**, 1169 (2019).

81. M. Schweizer, T. Jauffrais, C. Choquel, V. Méléder, S. Quinchard, E. Geslin, Trophic strategies of intertidal foraminifera explored with single-cell microbiome metabarcoding and morphological methods: What is on the menu? *Ecol. Evol.* **12**, e9437 (2022).

82. J. C. Varekamp, E. Thomas, F. Lugolobi, M. Buchholtz ten Brink, "The paleo-environmental history of Long Island Sound as traced by organic carbon, silica, and stable isotope/trace element studies in sediment cores," in *Proceedings of the 6th Biennial Long Island Sound Research Conference* (National Oceanic and Atmospheric Administration, 2002), pp. 109–113.

83. J. S. Bradshaw, Laboratory studies on the rate of growth of the foraminifer, *Streblus beccarii* (Linné) var. *tepida* (Cushman). *J. Paleo.* **31**, 1138–1147 (1957).

84. D. Schnitker, Ecotypic variation in *Ammonia beccarii* (Linne). *J. Foram. Res.* **4**, 217–223 (1974).

85. H. F. Thomas, “Final summary report of ‘The geologic framework of Southern New England: Vibracoring of Long Island Sound’” (Minerals Management Service, Department of the Interior, 1989).

86. J. R. Stone, J. P. Schafer, E. H. London, M. L. DiGiacomo-Cohen, R. S. Lewis, W. B. Thompson, “Quaternary Geologic Map of Connecticut and Long Island Sound Basin” (Report 2784, USGS, 2005).

87. M. Groner, E. Thomas, J. C. Varekamp, Radiocarbon studies of Long Island Sound sediments. *Eos Trans. Am. Geophys. Union* **85**, GC43A-04 (2004).

88. M. Groner, “The carbon cycle in Long Island Sound: Radiocarbon studies of carbonates,” thesis, Wesleyan University, Middletown, CT (2004).

89. J. C. Varekamp, E. Thomas, M. Groner, “The late Pleistocene–Holocene History of Long Island Sound,” in *Seventh Biennial LIS Research Conference Proceedings* (National Oceanic and Atmospheric Administration, 2005), pp. 27–32.

90. M. H. Bothner, P. W. Gill, B. B. Taylor, H. A. Karl, “Chemical and textural characteristics of sediments at an EPA reference site for dredged material on the continental slope SW of the Farallon Islands” (Open-File Report, USGS, 1997).

91. J. C. Varekamp, E. Thomas, K. Beuning, M. Buchholtz ten Brink, E. Mecray, “Environmental Change in Long Island Sound over the last 400 years” (EPA, 2004).

92. V. Paskevich, L. J. Poppe, “Georeferenced Sea-Floor Mapping and Bottom Photography in Long Island Sound” (USGS, 2000).

93. L. J. Poppe, V. F. Paskevich, “Geological framework data from Long Island Sound 1981-1990: A digital data release” (USGS, 2002).

94. E. Thomas, J. C. Varekamp, S. Cooper, F. Sangiorgi, T. Donders, “Microfossil proxies for anthropogenic environmental changes in Long Island Sound,” in *Abstracts with Programs* (Geological Society of America, 2010), vol. **42**, p. 175.

95. J. C. Varekamp, “Carbon cycling in LIS: Nutrient fluxes and landscape development over the last 1000 years,” in *Ninth Biennial LIS Research Conference Proceedings* (National Oceanic and Atmospheric Administration, 2009), pp. 2–13.
